# Thinking in Calories

**Published:** 1917-11-03

**Authors:** 


### THINKING IN CALORIES.

THE need for the most stringent economy in foodstuffs is greater than ever before, but it is extremely doubtful whether the *rationale* of food restriction has ever been made sufficiently clear to the British householder. In a little pamphlet issued by the Ministry of Food, Dr. Edmund Spriggs, M.D., F.R.C.P., explains in simple and non-technical language the precise food value of every common article of food, the amount of food required by all classes of persons, reckoned in calories, and how to cook many articles of food that are not generally appreciated owing to faulty preparation.

"The housewife," he says, "should not be afraid of the word 'calorie.' If the value of purchases is added up roughly in calories, the arithmetic will be found to be easier than that which is required to balance the weekly books. There is no one so experienced that a knowledge of food values will not help her to buy to greater advantage." Such terms, so bewildering to the lay mind, as proteins, carbo-hydrates, energy values, and the rest are accordingly explained, and their intimate connection with our physical well-being made clear.

The value of a pamphlet such as this depends very largely upon a judicious blend of theory with practice, and it adds greatly to the value of Dr. Spriggs' little book to find many simple recipes and hints for the preparation of economical dishes and substitutes for many of the commodities of which there is a shortage. Before the war English methods of cooking were the surprise and horror of many Continental housewives. The waste of fragments of food and the generally unappetising methods of preparation which then obtained must go by the board if we at home are to play our part in seconding the efforts of the men in the fighting line. The personal attention to detail and scrupulous observance of economy which is the rule in even the largest French households are features which our own housekeepers would do well to copy. For that reason, if for no other, every effort should be made by the Department concerned to popularise this and similar pamphlets, and not be content to let them fall into oblivion when once they have been published. The public have a somewhat natural dread of the Blue-book and the White Paper, and the make-up of these avowedly popular manuals should be as attractive as possible. Only recently the Department of Fisheries and Agriculture issued an admirable little pamphlet on the uses to which the fresh fish with which our rivers and streams abound could be put. Many succulent recipes were given for the cooking of such fish as pike, dace, and perch, which, as a rule, the angler throws away. There was much information in it of interest and importance to every fisherman. Yet few or none seem ever to have heard of its existence. The railway bookstalls certainly do not keep it in stock, nor did a number of bookshops at which we searched in vain. It is to be hoped that the present pamphlet will not share a like fate. Why should not a publication such as this, intended for popular consumption, be presented with some attractiveness? A picture cover should not be beyond the resources of the Government printers, and might assist materially in securing for it a large circulation.

